# Clinical Significance of Maternal Anti-D Antibody Titers in Severe RhD Alloimmunization Requiring Intrauterine Transfusion: A Tertiary Referral Center Experience

**DOI:** 10.3390/jcm15176825

**Published:** 2026-09-03

**Authors:** Serdar Aykut, İsmail Cüneyt Evrüke, Süleyman Cansun Demir, Emre Yalçın, Aslıhan Kurt, Ahmet Zeki Nessar

**Affiliations:** 1 Division of Perinatology, Department of Obstetrics and Gynecology, Mardin Training and Research Hospital, Mardin 47100, Türkiye; 2 Division of Perinatology, Department of Obstetrics and Gynecology, Cukurova University Faculty of Medicine, Adana 01330, Türkiye; cuneytevruke@gmail.com (İ.C.E.); cansundemir@gmail.com (S.C.D.); dremreyalcin23@gmail.com (E.Y.); 3 Division of Perinatology, University of Health Sciences, Gaziantep City Hospital, Gaziantep 27060, Türkiye; aslihanzeylikurt@gmail.com; 4 Division of Perinatology, Department of Obstetrics and Gynecology, Osmaniye State Hospital, Osmaniye 80000, Türkiye; zekinessar@gmail.com

**Keywords:** RhD alloimmunization, intrauterine transfusion, fetal anemia, hydrops fetalis, hemolytic disease of the fetus and newborn

## Abstract

**Background:** Maternal anti-D antibody titers are routinely used for risk stratification in RhD alloimmunization before the development of fetal anemia. However, their association with disease severity and perinatal outcomes among pregnancies that have already progressed to severe fetal anemia requiring intrauterine transfusion (IUT) remains unclear. This study aimed to investigate the association between maternal anti-D antibody titers, disease severity, and perinatal outcomes in pregnancies complicated by severe RhD alloimmunization requiring IUT. **Methods:** This retrospective cohort study included 38 RhD-alloimmunized pregnancies complicated by severe fetal anemia and managed with IUT at a tertiary referral center between January 2017 and December 2022. Maternal characteristics, anti-D antibody titers, presence of hydrops fetalis, IUT characteristics, delivery data, neonatal outcomes, and mortality were evaluated. Maternal anti-D antibody titers were analyzed in relation to disease severity and perinatal outcomes. **Results:** Hydrops fetalis was present in 24 fetuses (63.2%). The mean maternal age was 30.3 ± 4.8 years, and the mean gestational age at diagnosis was 23.5 ± 5.8 weeks. Maternal anti-D antibody titers were significantly higher in pregnancies complicated by hydrops fetalis than in non-hydropic pregnancies (*p* = 0.014). No significant associations were observed between maternal anti-D antibody titers and intrauterine fetal demise, neonatal mortality, neonatal intensive care unit admission, Apgar scores, or the number of IUT procedures. **Conclusions:** Among pregnancies with severe RhD alloimmunization requiring intrauterine transfusion, higher maternal anti-D antibody titers were associated with hydrops fetalis, suggesting an association with disease severity. However, no statistically significant associations were observed between maternal anti-D antibody titers and other evaluated perinatal outcomes in this high-risk cohort. These findings indicate that, once severe fetal anemia requiring IUT has developed, maternal anti-D antibody titers should be interpreted alongside fetal Doppler surveillance and comprehensive clinical assessment rather than as independent predictors of perinatal prognosis.

## 1. Introduction

Hemolytic disease of the fetus and newborn (HDFN) remains an important cause of fetal and neonatal morbidity and mortality despite the widespread implementation of anti-D immunoglobulin prophylaxis. The disease results from the transplacental passage of maternal IgG alloantibodies directed against fetal red blood cell antigens, leading to progressive fetal hemolysis, fetal anemia, hydrops fetalis, and, in severe cases, fetal or neonatal death. More than 50 maternal red blood cell antibodies have been implicated in HDFN; however, anti-D, anti-K (Kell), and anti-c antibodies account for most cases requiring fetal intervention. RhD alloimmunization continues to represent one of the leading causes of severe HDFN worldwide, particularly in pregnancies with inadequate prophylaxis, delayed diagnosis, or referral to tertiary fetal medicine centers [1,2,3,4].

RhD alloimmunization develops when an Rh-negative pregnant woman is exposed to RhD-positive fetal erythrocytes, most commonly through fetomaternal hemorrhage. Following sensitization, maternal IgG antibodies readily cross the placenta during subsequent pregnancies and may cause progressive fetal anemia. Untreated severe fetal anemia may progress to high-output cardiac failure, hydrops fetalis, and intrauterine fetal demise [2,5].

Over the past three decades, major advances in fetal medicine have dramatically improved the prognosis of pregnancies complicated by severe RhD alloimmunization. The introduction of middle cerebral artery peak systolic velocity (MCA-PSV) Doppler surveillance together with ultrasound-guided intrauterine transfusion (IUT) has substantially reduced fetal mortality. Contemporary multicenter studies have reported fetal survival rates exceeding 90% in non-hydropic fetuses and significantly improved outcomes even among hydropic fetuses when timely referral and treatment are achieved [6,7,8,9]. Accordingly, current international clinical practice guidelines recommend integrated management based on maternal antibody screening, fetal antigen determination when available, serial MCA-PSV Doppler surveillance, and timely IUT in pregnancies at risk for severe fetal anemia [7].

Maternal anti-D antibody titers remain an essential component of the initial assessment of RhD alloimmunization and are widely used to identify pregnancies requiring intensified fetal surveillance. Previous studies have demonstrated that higher maternal antibody titers are associated with an increased likelihood of severe fetal anemia, hydrops fetalis, intrauterine transfusion, and postnatal treatment requirements. However, once pregnancies progress to severe fetal anemia requiring intrauterine transfusion, it remains uncertain whether maternal anti-D antibody titers continue to reflect disease severity or provide additional prognostic information regarding perinatal outcomes within this selected high-risk population [8,9,10].

The aim of this study was not to reassess the established role of maternal anti-D antibody titers in screening for fetal anemia, but to explore their prognostic value within a highly selected cohort of pregnancies that had already progressed to severe RhD alloimmunization requiring IUT. Specifically, we investigated whether maternal anti-D titers at referral were associated with hydrops fetalis and subsequent fetal and neonatal outcomes in this advanced stage of disease.

## 2. Materials and Methods

### 2.1. Study Design and Patient Selection

This retrospective cohort study was conducted at Cukurova University Faculty of Medicine, a tertiary fetal therapy referral center in southern Türkiye that serves as a regional referral center for pregnancies complicated by severe fetal anemia requiring intrauterine transfusion (IUT). Medical records of all consecutive pregnant women with RhD alloimmunization who underwent IUT at our institution between January 2017 and December 2022 were retrospectively reviewed. After application of the predefined eligibility criteria, 38 pregnancies with fetal anemia secondary to RhD alloimmunization constituted the final study cohort. Pregnancies complicated by fetal anemia due to non-Rh red blood cell alloimmunization, parvovirus B19 infection, fetomaternal hemorrhage, twin-to-twin transfusion syndrome, or twin anemia-polycythemia sequence were excluded. No additional cases were excluded after application of the predefined eligibility criteria.

As a tertiary referral center, our institution receives patients from multiple surrounding provinces. Women referred with established RhD alloimmunization underwent prompt maternal immunohematological evaluation and fetal assessment immediately after admission. Maternal anti-D antibody titers were determined in the Immunohematology Laboratory using the indirect antiglobulin test (IAT) with the conventional tube agglutination assay. In pregnancies referred because of suspected severe fetal anemia, fetal ultrasonography and Doppler evaluation were performed without unnecessary delay, and intrauterine transfusion was undertaken as soon as fetal anemia was confirmed. For statistical analyses, the maternal anti-D antibody titer obtained at presentation to our tertiary referral center was used. Maternal antibody titers were analyzed as continuous variables without predefined titer categories.

Pregnancies referred before the development of fetal anemia were managed according to our institutional surveillance protocol. In the absence of hydrops fetalis and with maternal anti-D antibody titers ≤1:16, fetal surveillance with middle cerebral artery peak systolic velocity (MCA-PSV) Doppler ultrasonography was initiated at 18 weeks of gestation. Patients with MCA-PSV values <1.5 MoM were monitored every two weeks. Fetuses with MCA-PSV values ≥1.5 MoM were considered to have suspected severe fetal anemia. Fetal blood sampling by cordocentesis was routinely performed immediately before intrauterine transfusion to confirm fetal anemia and determine the pre-transfusion fetal hemoglobin and hematocrit levels. Pre-transfusion fetal hemoglobin and hematocrit values obtained during fetal blood sampling were retrospectively retrieved from the electronic medical records whenever available. Because historical laboratory records were incomplete, these measurements were available for only 14 of the 38 fetuses (36.8%). In our institution, intrauterine transfusion was performed when fetal blood sampling confirmed moderate-to-severe fetal anemia based on gestational age-adjusted fetal hemoglobin values, together with the overall clinical assessment including MCA-PSV findings. Before transfusion, all fetuses at or beyond 24 weeks of gestation received antenatal corticosteroid prophylaxis consisting of two intramuscular doses of 12 mg betamethasone administered 24 h apart. Intravenous magnesium sulfate was not administered routinely before all intrauterine transfusion procedures. Instead, it was administered according to our institutional protocol only when preterm delivery was considered likely and fetal neuroprotection was indicated.

The primary outcome of the study was the association between maternal anti-D antibody titers and hydrops fetalis. Secondary outcomes included intrauterine fetal demise, neonatal mortality, gestational age at delivery, birth weight, neonatal intensive care unit admission, Apgar scores, postnatal transfusion requirements, and the number of intrauterine transfusions.

Maternal demographic characteristics, gestational age at presentation, maternal anti-D antibody titers, MCA-PSV measurements, estimated fetal weight, presence of hydrops fetalis, transfused blood volume, number of IUT procedures, pre- and post-transfusion fetal hemoglobin levels, gestational age at delivery, birth weight, mode of delivery, neonatal outcomes, and procedure-related complications were retrieved from the electronic medical records.

This study was conducted in accordance with the principles of the Declaration of Helsinki and was approved by the Ethics Committee of Cukurova University Faculty of Medicine (Approval No. 134; 2 June 2023). The requirement for informed consent was waived because of the retrospective design of the study.

### 2.2. IUT Procedure

All intrauterine transfusion procedures were performed by at least one maternal–fetal medicine specialist and/or fellow, assisted by a nurse experienced in intrauterine transfusion procedures. The transfusion site was selected according to the operator’s preference based on fetal position and placental localization on the uterine wall. Intrauterine transfusions were administered into the umbilical vein either at the placental cord insertion site or through a free loop of the umbilical cord. The volume of blood to be transfused was calculated according to the formula proposed by Giannina et al. in 1998, as follows [11]:

Transfusion volume (mL) = fetoplacental blood volume (mL) × [(target hematocrit − fetal hematocrit)/donor hematocrit]

Fetoplacental blood volume (mL) = 1.046 + (estimated fetal weight × 0.14)

Transfused blood products consisted of type O Rh-negative packed red blood cells cross-matched with maternal erythrocytes, negative for cytomegalovirus, leukocyte-depleted, irradiated, and concentrated to a hematocrit level of 75–80%. Blood units stored for no longer than 5 days were preferred.

The timing of the second intrauterine transfusion was determined according to post-transfusion fetal hemoglobin levels and MCA Doppler assessment. The expected decline in fetal hemoglobin was calculated at a rate of 0.4 g/dL/day to estimate the timing of the subsequent transfusion [12]. We did not use MCA PSV evaluation for predicting anemia after the second transfusion and the timing of third and subsequent transfusion was based on the expected decline in fetal hemoglobin of 0.3 g/dL/day and 0.2 g/dL/day after the second or third transfusion, respectively [12,13].

### 2.3. Statistical Analysis

Statistical analyses were performed using IBM SPSS Statistics for Windows, version 25.0 (IBM Corp., Armonk, NY, USA). Continuous variables were expressed as mean ± standard deviation (SD) or median (minimum–maximum), as appropriate, whereas categorical variables were presented as frequencies and percentages. The normality of continuous variables was assessed using the Shapiro–Wilk test.

Because maternal anti-D antibody titers were not normally distributed, nonparametric statistical methods were used for analyses involving antibody titers. Given the wide range of maternal anti-D antibody titers, logarithmic transformation was considered. However, the primary analyses involving antibody titers were performed using rank-based nonparametric methods (Mann–Whitney U test and Kendall’s τ correlation), which are invariant to monotonic transformations. Therefore, logarithmic transformation would not alter the ranking of observations or the resulting statistical conclusions, and antibody titers were analyzed on their original scale. Differences in maternal anti-D antibody titers between independent groups were evaluated using the Mann–Whitney U test. Associations between categorical variables were assessed using the Pearson chi-square test or Fisher’s exact test, as appropriate; Fisher’s exact test was used when expected cell counts were less than 5. Correlations between maternal anti-D antibody titers and continuous clinical variables, including the number of intrauterine transfusions and Apgar scores, were evaluated using Kendall’s τ correlation coefficient. All statistical tests were two-tailed, and a *p*-value < 0.05 was considered statistically significant.

## 3. Results

A total of 38 pregnancies complicated by RhD alloimmunization and managed with intrauterine transfusion were included in the study. Baseline clinical characteristics and perinatal outcomes of the study population are summarized in Table 1.

Hydrops fetalis was present in 24 fetuses (63.2%), whereas 14 fetuses (36.8%) had no evidence of hydrops. Regarding transfusion requirements, 11 fetuses (28.9%) underwent a single intrauterine transfusion, 17 (44.7%) underwent two procedures, 5 (13.2%) underwent three procedures, 3 (7.9%) underwent four procedures, and 2 fetuses (5.3%) required five transfusions.

Delivery was performed by cesarean section in 34 cases (89.5%), while 4 pregnancies (10.5%) resulted in vaginal delivery. Eighteen neonates (47.4%) were male and 20 (52.6%) were female. Postnatal phototherapy was required in 11 neonates (28.9%), erythrocyte suspension transfusion in 13 (34.2%), and exchange transfusion in 6 neonates (18.7%). Overall, 17 neonates (44.7%) required at least one postnatal therapeutic intervention.

Among hydropic fetuses, intrauterine fetal demise occurred in 6 cases, while neonatal death occurred in 3 of the liveborn neonates.

Maternal age ranged between 18 and 40 years, with a mean age of 30.26 ± 4.80 years. Gestational age at presentation ranged from 7 to 31 weeks, with a mean gestational age of 23.47 ± 5.78 weeks. Descriptive statistics of the maternal, fetal, and neonatal variables analyzed in the study are presented in Table 2.

Pre-transfusion fetal hemoglobin and hematocrit measurements were available for 14 of the 38 fetuses (36.8%), whereas these measurements could not be retrieved for the remaining 24 cases (63.2%) because of incomplete historical laboratory records. Among the available cases, the median pre-transfusion fetal hemoglobin concentration was 4.35 g/dL (IQR, 3.48–6.28), and the median pre-transfusion hematocrit was 12.95% (IQR, 12.45–16.73). In an exploratory complete-case analysis, pre-transfusion fetal hemoglobin levels were significantly lower in hydropic fetuses than in non-hydropic fetuses (median 3.70 vs. 8.20 g/dL, *p* = 0.006), whereas the difference in pre-transfusion hematocrit did not reach statistical significance (median 12.70% vs. 23.60%, *p* = 0.056). Because these analyses were based on a limited number of available cases, the findings should be interpreted cautiously (Supplementary Table S1). To assess the potential for selection bias related to missing pre-transfusion laboratory data, baseline and relevant clinical characteristics were compared between fetuses with available (*n* = 14) and unavailable (*n* = 24) pre-transfusion hemoglobin and hematocrit measurements. No statistically significant differences were observed between the groups in maternal age, gestational age at presentation, maternal anti-D antibody titer, presence of hydrops fetalis, number of IUT procedures, gestational age at delivery, birth weight, or intrauterine fetal demise (all *p* > 0.05; Supplementary Table S2). Comparative analyses were performed to assess differences in maternal anti-D antibody titers according to various perinatal outcome variables. Because antibody titers did not demonstrate normal distribution based on the Shapiro–Wilk test (*p* < 0.001), non-parametric statistical methods were used. Accordingly, intergroup comparisons were conducted using the Mann–Whitney U test.

Maternal anti-D antibody titers did not differ significantly according to neonatal intensive care unit admission status (*p* = 0.852), intrauterine fetal demise (*p* = 0.888), neonatal mortality (*p* = 0.160), overall survival status without intrauterine or neonatal death (*p* = 0.175), or 5 min Apgar scores (*p* = 0.288). However, a statistically significant difference in maternal antibody titers was observed between hydropic and non-hydropic fetuses (*p* = 0.014), with significantly higher antibody titers detected in pregnancies complicated by hydrops fetalis. Detailed comparisons of maternal anti-D antibody titers according to perinatal outcomes are presented in Table 3.

A chi-square test of independence was performed to evaluate the association between the presence of hydrops fetalis and intrauterine fetal demise. All non-hydropic fetuses survived without intrauterine demise, whereas intrauterine fetal demise occurred in 6 of the 24 hydropic fetuses. However, the association between hydrops fetalis and intrauterine fetal demise did not reach statistical significance according to chi-square analysis (*p* = 0.067). The association between hydrops fetalis and intrauterine fetal demise is detailed in Table 4.

Because maternal anti-D antibody titers did not demonstrate normal distribution, Kendall’s Tau correlation analysis was performed to evaluate the relationship between antibody titers and the number of intrauterine transfusions, as well as 1 min and 5 min Apgar scores. Correlation analysis revealed no statistically significant association between maternal anti-D antibody titers and the number of intrauterine transfusions (*p* = 0.409), 1 min Apgar scores (*p* = 0.516), or 5 min Apgar scores (*p* = 0.683). Correlation analyses between maternal anti-D antibody titers, IUT requirement, and Apgar scores are detailed in Table 5.

Procedure-related complications occurred in two intrauterine transfusion procedures, both consisting of umbilical cord hematomas. Neither complication resulted in immediate fetal demise during the procedure.

## 4. Discussion

In this retrospective cohort study, we evaluated the association between maternal anti-D antibody titers and prenatal as well as postnatal outcomes in RhD-alloimmunized pregnancies complicated by fetal anemia requiring intrauterine transfusion. The principal finding of the study was that elevated maternal anti-D antibody titers were significantly associated with the presence of hydrops fetalis. In contrast, maternal antibody titers were not significantly associated with neonatal mortality, intrauterine fetal demise, duration of neonatal intensive care unit stay, number of intrauterine transfusions, or Apgar scores. These findings suggest that higher maternal anti-D antibody titers are associated with the presence of hydrops fetalis and may reflect greater disease severity within this highly selected cohort. However, maternal antibody titers alone did not demonstrate clear prognostic value for the evaluated perinatal outcomes in this cohort. However, given the small sample size and limited number of adverse outcome events, the absence of statistically significant associations should not be interpreted as evidence of no association, as insufficient statistical power may have limited our ability to detect clinically relevant relationships.

Recent studies have suggested that maternal anti-D antibody titers may be associated with the severity of fetal and neonatal disease in RhD alloimmunization. In a retrospective study published in 2024, Tang et al. evaluated 142 RhD-sensitized pregnancies and stratified patients into low-, moderate-, and high-titer groups according to maternal anti-D antibody levels [10]. The authors reported that pregnancies in the high-titer group had increased requirements for intrauterine transfusion, a higher number of transfusion procedures, and greater need for neonatal top-up and exchange transfusions. However, the frequency of major neonatal complications did not differ significantly among the groups. Our findings are partially consistent with those reported by Tang et al. Both studies indicate that higher maternal anti-D antibody titers are associated with greater disease severity. Nevertheless, important methodological and clinical differences should be considered when interpreting these findings. Tang et al. evaluated the entire spectrum of RhD-sensitized pregnancies using predefined antibody titer categories, whereas our study was limited to pregnancies that had already progressed to severe fetal anemia requiring intrauterine transfusion and analyzed maternal anti-D antibody titers as continuous variables. Furthermore, Tang et al. demonstrated an association between higher antibody titers and an increased number of intrauterine transfusions, whereas no statistically significant association between maternal anti-D antibody titers and the number of IUT procedures was observed in our cohort. This discrepancy is most likely explained by differences in study populations, disease severity at inclusion, and analytical approaches rather than conflicting biological effects of maternal anti-D antibody titers [10].

In the present study, maternal anti-D antibody titers were significantly higher in pregnancies complicated by hydrops fetalis compared with non-hydropic cases. Pre-transfusion fetal hemoglobin and hematocrit measurements were available for only 14 of the 38 fetuses included in the study. In an exploratory complete-case analysis, hydropic fetuses had significantly lower pre-transfusion fetal hemoglobin concentrations than non-hydropic fetuses, whereas the difference in hematocrit did not reach statistical significance. These findings are biologically consistent with the established pathophysiology of severe fetal anemia. However, because these analyses were based on a limited subset of patients with available historical laboratory records, they should be interpreted cautiously and regarded as hypothesis-generating rather than confirmatory. This finding suggests that increased maternal antibody burden in alloimmune hemolytic disease may contribute to progressive fetal erythrocyte destruction, severe fetal anemia, and ultimately the development of hydrops fetalis. Nevertheless, the pathogenesis of hydrops is not solely dependent on maternal antibody titers. Multiple additional factors, including the biological activity of the antibody, fetal antigen expression, transplacental antibody transfer, fetal erythropoietic response, and gestational age at diagnosis, may influence disease severity and clinical presentation. Therefore, although maternal antibody titers remain important for risk stratification, MCA-PSV Doppler surveillance is currently considered the cornerstone for the assessment of fetal anemia. Consistent with our findings, a recent 2025 clinical practice guideline emphasized that the management of pregnancies complicated by red blood cell alloimmunization should incorporate integrated evaluation of maternal antibody titers, fetal antigen status, and MCA-PSV Doppler monitoring [7].

In our cohort, maternal anti-D antibody titers were not significantly associated with the number of intrauterine transfusions performed. This observation is likely related to the highly selected nature of our study population, which consisted exclusively of pregnancies requiring intrauterine transfusion for severe fetal anemia. Consequently, the narrower and more advanced disease spectrum may have attenuated the relationship between maternal anti-D antibody titers and transfusion requirements. Moreover, the number of intrauterine transfusions is influenced not only by maternal antibody burden but also by several additional clinical factors, including gestational age at initial presentation, baseline fetal hemoglobin level, the presence of hydrops fetalis, post-transfusion hematocrit response, and the interval between transfusion and delivery.

The prognostic impact of hydrops fetalis has been strongly emphasized in the literature. In an 18-year tertiary referral center experience published by Pan et al. in 2023 [6], including pregnancies managed with intrauterine transfusion for severe alloimmunization, IUT was reported to be an effective and safe therapeutic approach; however, early recognition and timely treatment of hydrops fetalis were highlighted as critical determinants of favorable perinatal outcomes. Consistent with these observations, intrauterine fetal demise in our cohort occurred exclusively among hydropic fetuses, whereas all non-hydropic fetuses survived throughout the perinatal period. Although the association between hydrops fetalis and intrauterine fetal demise did not reach statistical significance, this finding remains clinically relevant. The lack of statistical significance may reflect the limited sample size and consequent reduction in statistical power. Alternatively, hydrops fetalis may not have been independently associated with intrauterine fetal demise within this highly selected cohort of pregnancies requiring intrauterine transfusion [6]. Compared with the recent international DIONYSOS collaborative study, our cohort demonstrated substantially higher rates of hydrops fetalis and perinatal mortality. The DIONYSOS study, which included pregnancies managed with intrauterine transfusion from 31 participating centers across multiple countries, reported considerably lower fetal mortality than observed in our series. This discrepancy is most likely explained by differences in referral patterns and case selection rather than treatment strategy. Cukurova University serves as a major tertiary fetal therapy referral center in southern Türkiye, receiving complicated pregnancies from a large geographic region. Consequently, many patients are referred only after severe fetal anemia has already developed, and a considerable proportion already presented with hydrops fetalis at the time of admission. Although the exact interval between the diagnosis of fetal anemia and referral could not be determined because of the retrospective design of the study, the high prevalence of hydrops at presentation is consistent with referral after progression to advanced fetal disease in a substantial proportion of cases. Therefore, the relatively poor perinatal outcomes observed in our cohort should be interpreted within the context of a highly selected population with advanced disease rather than as representative of all pregnancies affected by RhD alloimmunization managed with intrauterine transfusion.

In our series, procedure-related complications were uncommon, with only two cases of umbilical cord hematoma recorded during intrauterine transfusion, suggesting that the high perinatal mortality was unlikely to be explained by procedural complications alone.

In the present study, maternal anti-D antibody titers were not significantly associated with neonatal mortality, duration of neonatal intensive care unit stay, or Apgar scores. These findings suggest that neonatal prognosis in RhD alloimmunization is not determined solely by antenatal antibody titers. Several additional factors, including fetal hemoglobin levels following intrauterine transfusion, gestational age at delivery, degree of prematurity, birth weight, resolution of hydrops fetalis, and the availability of advanced neonatal intensive care support, play critical roles in determining neonatal outcomes. Similarly, recent intrauterine transfusion series published in 2024 reported that anti-D antibodies remain the most common cause of severe fetal hemolysis; however, perinatal prognosis appears to be strongly influenced by the timing of transfusion, the presence of hydrops fetalis, and institutional experience in the management of severe alloimmunization [9].

An important consideration regarding the clinical utility of maternal antibody titers in contemporary practice is that, although antibody levels remain valuable during the initial risk assessment of RhD alloimmunization, their utility as standalone monitoring parameters is limited once fetal anemia develops or the pregnancy enters the intrauterine transfusion stage. In their 2020 update, Castleman et al. emphasized that the modern management of red blood cell alloimmunization relies not only on maternal serologic assessment but also on noninvasive Doppler surveillance of fetal anemia and timely intrauterine intervention when indicated [2]. In this context, the findings of the present study support the concept that elevated maternal anti-D antibody titers may serve as a marker of increased disease severity; however, antibody titers alone should not be considered sufficient for prognostic assessment of intrauterine transfusion requirements, neonatal mortality, or postnatal therapeutic needs [2]. The present study suggests that, among pregnancies already requiring intrauterine transfusion, maternal anti-D antibody titers provide limited additional prognostic information beyond contemporary fetal surveillance. Although elevated antibody titers were associated with the presence of hydrops fetalis, no statistically significant associations were observed between maternal anti-D antibody titers and most other adverse perinatal outcomes evaluated in this cohort. Therefore, once severe fetal anemia has developed, clinical decision-making should rely primarily on fetal condition, including MCA-PSV Doppler assessment and overall clinical evaluation, rather than maternal anti-D antibody titers alone.

This study has several limitations. First, its retrospective design and relatively small sample size may limit the statistical power for detecting associations with rare adverse outcomes. Second, because only pregnancies requiring intrauterine transfusion were included, the findings may not be generalizable to all RhD-alloimmunized pregnancies. Additionally, serial maternal anti-D antibody measurements and detailed obstetric history, including parity, previous affected pregnancies, and prior intrauterine transfusions, were not consistently available because many patients were referred after alloimmunization had already been diagnosed and managed at external institutions. In addition, pre-transfusion fetal hemoglobin and hematocrit measurements obtained by cordocentesis were available for only 14 of the 38 fetuses (36.8%), whereas these data could not be retrieved for the remaining 24 cases because of incomplete historical laboratory records. Although exploratory complete-case analyses were performed using the available measurements, the substantial proportion of missing data introduces the possibility of selection bias and limits the robustness and generalizability of these findings. Although no statistically significant differences were identified in the evaluated baseline and clinical characteristics between cases with and without available pre-transfusion laboratory measurements, selection bias cannot be excluded given the small sample size and substantial proportion of missing data. Therefore, the results of the complete-case analyses should be interpreted with caution. Consequently, longitudinal changes in maternal antibody titers and the potential influence of previous obstetric history on disease severity could not be evaluated. Furthermore, the broad gestational age range at presentation introduced clinical heterogeneity that could not be explored in subgroup analyses because of the limited sample size. Moreover, the relatively small sample size and the limited number of outcome events precluded reliable multivariable analyses to adjust for potential confounding factors. Consequently, it was not possible to determine whether maternal anti-D antibody titers were independently associated with hydrops fetalis or other perinatal outcomes. Therefore, the observed associations should be interpreted as exploratory rather than confirmatory.

## 5. Conclusions

In conclusion, elevated maternal anti-D antibody titers in RhD-alloimmunized pregnancies requiring intrauterine transfusion were significantly associated with the presence of hydrops fetalis, suggesting an association with greater disease severity within this highly selected cohort. However, no statistically significant associations were observed between maternal anti-D antibody titers and neonatal mortality, intrauterine fetal demise, postnatal treatment requirements, or Apgar scores in this cohort. Although maternal anti-D antibody titers remain an established component of the clinical assessment of RhD alloimmunization, their role in the initial assessment was not directly evaluated in the present study. Our findings suggest that, once severe fetal anemia requiring intrauterine transfusion has developed, clinical decision-making should rely primarily on comprehensive fetal assessment, particularly MCA-PSV Doppler surveillance, together with individualized fetal and neonatal management. Given the retrospective design, small sample size, and highly selected study population, these findings should be considered exploratory and require confirmation in larger, preferably multicenter cohorts.

## Figures and Tables

**Table 1:** Baseline Clinical Characteristics and Perinatal Outcomes of RhD-Alloimmunized Pregnancies.

Variable	*n* (%)
Hydrops fetalis	
Absent	14 (36.8)
Present	24 (63.2)
Number of Intrauterine Transfusions (IUTs)	
1 procedure	11 (28.9)
2 procedures	17 (44.7)
3 procedures	5 (13.2)
4 procedures	3 (7.9)
5 procedures	2 (5.3)
Mode of Delivery	
Vaginal delivery	4 (10.5)
Cesarean delivery	34 (89.5)
Fetal Sex	
Male	18 (47.4)
Female	20 (52.6)
Phototherapy Requirement	
No	27 (71.1)
Yes	11 (28.9)
Erythrocyte Suspension Transfusion	
No	25 (65.8)
Yes	13 (34.2)
Exchange Transfusion	
No	26 (81.3)
Yes	6 (18.7)
Postnatal Treatment Requirement	
No	21 (55.3)
Yes	17 (44.7)

**Table 2:** Descriptive Statistics of Maternal, Fetal, and Neonatal Variables.

Variable	Mean ± SD	Median (Minimum–Maximum)
Maternal age (years)	30.26 ± 4.80	31 (18–40)
Maternal anti-D antibody titer *	—	8192 (64–524,288)
Gestational age at presentation (weeks)	23.47 ± 5.78	25 (7–31)
Number of intrauterine transfusions (IUTs)	2.16 ± 1.10	2 (1–5)
Gestational age at delivery (weeks)	31.00 ± 3.73	31.5 (23–39)
Birth weight (g)	1812.24 ± 738.75	1875 (528–3870)
1 min Apgar score	4.34 ± 2.67	4.5 (0–9)
5 min Apgar score	6.16 ± 3.10	7 (0–10)
Duration of NICU stay (days)	10.95 ± 16.29	1.5 (0–60)

Abbreviations: SD, standard deviation; IUT, intrauterine transfusion; NICU, neonatal intensive care unit. Values are presented as minimum, maximum, mean, and standard deviation. * Maternal anti-D antibody titers demonstrated non-normal distribution according to the Shapiro–Wilk test (*p* < 0.001); therefore, median and range values are presented.

**Table 3:** Comparison of Maternal Anti-D Antibody Titers According to Fetal and Perinatal Outcomes.

Variable	Group	*n*	Maternal Anti-D Antibody Titer, Median (IQR)	Mann–Whitney U	*p* Value
NICU admission *	No	7	8192 (6144–65,536)	92	0.852
	Yes	25	16,384 (4096–32,768)		
Hydrops fetalis	Absent	14	6144 (2048–16,384)	87.5	0.014
	Present	24	32,768 (8192–65,536)		
Neonatal mortality	No	28	12,288 (4096–32,768)	98	0.160
	Yes	10	32,768 (14,336–57,344)		
Intrauterine fetal demise	No	32	16,384 (4096–40,960)	92.5	0.888
	Yes	6	16,384 (10,240–28,672)		
Overall survival	Survived	22	12,288 (4096–32,768)	130.5	0.175
	Died	16	32,768 (8192–40,960)		
5 min Apgar score **	≤7	18	32,768 (8192–32,768)	156	0.288
	≥8	14	8192 (2560–53,248)		

Data are presented as median (interquartile range [IQR]). Comparisons between groups were performed using the Mann–Whitney U test because maternal anti-D antibody titers were not normally distributed. * Analysis was restricted to live-born infants. NICU admission was defined as a NICU stay of >0 days. ** Analysis was restricted to live-born infants.

**Table 4:** Association Between Hydrops Fetalis and Intrauterine Fetal Demise.

Hydrops Fetalis	No IUFD	IUFD	*p* Value
Absent	14	0	
Present	18	6	0.067

Fisher’s exact test was used for statistical analysis.

**Table 5:** Correlation of Maternal Anti-D Antibody Titers with IUT Requirement and Apgar Scores.

Variable	Correlation Coefficient (τ)	*p* Value
Number of intrauterine transfusions (IUTs)	−0.109	0.409
1 min Apgar score	−0.089	0.516
5 min Apgar score	−0.060	0.683

Correlation analysis was performed using Kendall’s Tau Correlation coefficient (τ).

## Data Availability

The data that support the findings of this study are available from the corresponding author upon reasonable request.

## Supplementary Materials

The following supporting information can be downloaded at: https://www.mdpi.com/article/10.3390/jcm15176825/s1, Supplementary Table S1. Exploratory analysis of available pre-transfusion fetal hemoglobin and hematocrit variables. Data are presented as median (interquartile range [IQR]). Supplementary Table S2. Comparison of Baseline and Clinical Characteristics Between Cases With and Without Available Pre-transfusion Fetal Hemoglobin and Hematocrit Measurements.
