# Supplementary material for: Clinical Significance of Maternal Anti-D Antibody Titers in Severe RhD Alloimmunization Requiring Intrauterine Transfusion: A Tertiary Referral Center Experience
Source: J Clin Med. 2026 Sep 3;15(17):6825. doi: 10.3390/jcm15176825 (PMC13566301; doi:10.3390/jcm15176825)
Supplement: Supplementary file 1 [file jcm-15-06825-s001.zip › jcm-4391735-supplementary.pdf]

Supplementary Table S1. Exploratory analysis of available pre-transfusion fetal hemoglobin and hematocrit variables. Data are presented as median (interquartile range [IQR]).

**Variable**	**Overall (n = 14)**	**Hydrops fetalis (n = 10)**	**No hydrops (n = 4)**	**p value***
Pre-transfusion fetal hemoglobin (g/dL)	4.35 (3.48–6.28)	3.70 (3.40–4.58)	8.20 (7.30–9.13)	0.006
Pre-transfusion fetal hematocrit (%)	12.95 (12.45–16.73)	12.70 (11.50–14.48)	23.60 (18.30–27.75)	0.056

* Comparisons were performed using the Mann–Whitney U test.

Data are presented as median (interquartile range [IQR]). Pre-transfusion fetal hemoglobin and hematocrit measurements were available for 14 of the 38 fetuses (36.8%). Because analyses were based on complete cases only, these findings should be interpreted as exploratory.

Supplementary Table S2

Comparison of Baseline and Clinical Characteristics Between Cases With and Without Available Pre-transfusion Fetal Hemoglobin and Hematocrit Measurements

**Characteristic**	**Available Hb/Hct (n = 14)**	**Unavailable Hb/Hct (n = 24)**	**p value**
Maternal age (years)	31.0 (26.25–33.75)	31.0 (28.00–33.25)	0.927
Gestational age at presentation (weeks)	26.0 (22.75–27.75)	24.0 (19.00–28.25)	0.439
Maternal anti-D antibody titer	16,384 (5,120–32,768)	16,384 (4,096–65,536)	0.680
Hydrops fetalis, n (%)	10 (71.4)	14 (58.3)	0.501
Number of IUT procedures	2.0 (1.00–2.75)	2.0 (1.75–2.25)	0.641
Gestational age at delivery (weeks)	31.0 (29.00–32.00)	32.0 (28.75–34.25)	0.354
Birth weight (g)	1,500 (1,258.75–2,175)	2,017.5 (1,393.75–2,398.75)	0.155
Intrauterine fetal demise, n (%)	2 (14.3)	4 (16.7)	1.000

Data are presented as median (interquartile range) unless otherwise indicated. Continuous variables were compared using the Mann–Whitney U test, and categorical variables were compared using Fisher's exact test. Hb, hemoglobin; Hct, hematocrit; IUT, intrauterine transfusion.

No statistically significant differences were observed between cases with and without available pre-transfusion Hb/Hct measurements for the evaluated characteristics (all p > 0.05).
